# Coordinated Movements Prevent Jamming in an Emperor Penguin Huddle

**DOI:** 10.1371/journal.pone.0020260

**Published:** 2011-06-01

**Authors:** Daniel P. Zitterbart, Barbara Wienecke, James P. Butler, Ben Fabry

**Affiliations:** 1 Department of Physics, University of Erlangen-Nuremberg, Erlangen, Germany; 2 Alfred Wegener Institute for Polar and Marine Research (AWI), Bremerhaven, Germany; 3 Australian Antarctic Division, Kingston, Australia; 4 Molecular Integrative Physiological Sciences Program, Harvard School of Public Health, Boston, Massachusetts, United States of America; 5 Division of Sleep Medicine, Department of Medicine, Harvard Medical School and Brigham and Women's Hospital, Boston, Massachusetts, United States of America; University of Maribor, Slovenia

## Abstract

For Emperor penguins (***Aptenodytes forsteri***), huddling is the key to survival during the Antarctic winter. Penguins in a huddle are packed so tightly that individual movements become impossible, reminiscent of a jamming transition in compacted colloids. It is crucial, however, that the huddle structure is continuously reorganized to give each penguin a chance to spend sufficient time inside the huddle, compared with time spent on the periphery. Here we show that Emperor penguins move collectively in a highly coordinated manner to ensure mobility while at the same time keeping the huddle packed. Every 30–60 seconds, all penguins make small steps that travel as a wave through the entire huddle. Over time, these small movements lead to large-scale reorganization of the huddle. Our data show that the dynamics of penguin huddling is governed by intermittency and approach to kinetic arrest in striking analogy with inert non-equilibrium systems, including soft glasses and colloids.

## Introduction

Emperor penguins are the only vertebrates that breed during the austral winter where they have to endure temperatures below −45°C and winds of up to 50 m/s while fasting. From their arrival at the colony until the eggs hatch and the return of their mates, the males, who solely incubate the eggs, fast for about 110–120 days [1]–[3]. To conserve energy and to maintain their body temperature[4], the penguins aggregate in huddles where ambient temperatures are above 0°C and can reach up to 37°C [1]–[3].

Huddling poses an interesting physical problem. If the huddle density is too low, the penguins lose too much energy. If the huddle density is too high, internal rearrangement becomes impossible, and peripheral penguins are prevented to reach the warmer huddle center. This problem is reminiscent of colloidal jamming during a fluid-to-solid transition [5]. In this paper we show that Emperor penguins prevent jamming by a recurring short-term coordination of their movements.

## Materials and Methods

**Figure 1:**
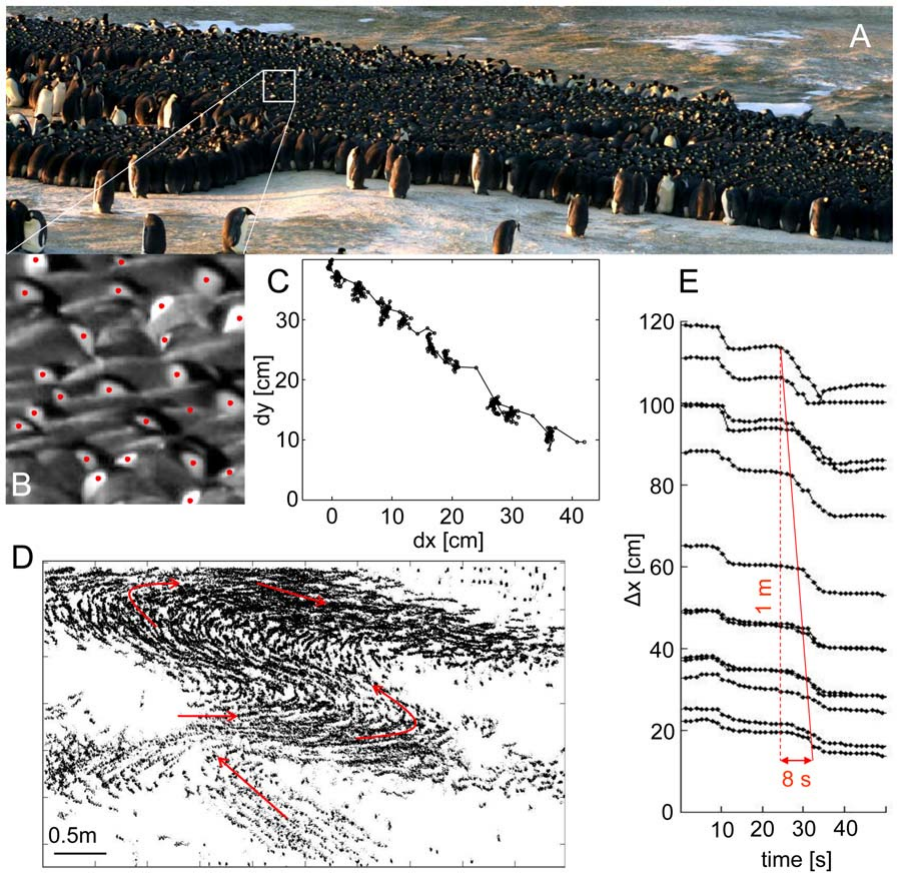
Coordinated movements in an emperor penguin huddle. (A) Observed field of view of the emperor penguin colony. The image shows several huddles and individual penguins. The density of penguins in huddles is approximately 21 animals per square meter. (B) The penguins' yellow and white face patch was used to track individual animals. (C) Typical trajectory of a penguin during huddle movements. Motionless periods are interrupted by intermittent small steps that lead over time to a reorganization of the entire huddle. (D) Positions of penguins tracked over 4 hours show a collective huddle movement as indicated by red arrows (movies available online). (E) Trajectories from neighboring penguins with similar vertical (y) positions show correlated steps in the horizontal (x) direction. The speed of the propagating wave is indicated by the slope of the red line.

To study positional reorganization processes in a penguin huddle, we observed a medium-size Emperor penguin colony (∼2000 animals) (Fig 1A, Movie S1) near the Neumayer Antarctic Research Station (70°39S 8°15W). From an elevated (12 m), distant (115 m) position, high resolution time lapse images were recorded every 1.3 sec for a total of 4 h and analyzed off-line to detect and track penguin positions (Fig 1B). The air temperatures during the recordings (03. Aug. 2008) varied from −33 to −43°C with a maximum wind speed of 8.3 m/s. Most penguins carried an egg. We did not observe any hatched chicks at that time, and the female penguins had not yet returned to the colony. The minimum distance of the investigators to the nearest penguins was greater than 100 m at all times, in accordance with the guidelines of the German Environmental Protection Agency. No specific ethical review was required for this study.

## Results

Emperor penguins have developed a surprising strategy to prevent jamming while remaining in a densely packed configuration. As the sun sets and the temperatures drop from −33 to −43°C in the course of the recording, the penguins aggregate in multiple huddles. For most of the time, these huddles remain motionless, with the penguins tightly packed. Penguins in any given huddle face in the same direction, but because of the low wind speeds during the time of the recording, this direction is not dictated by the wind direction and differs between huddles [5].

The jammed state of the huddle is interrupted every 30–60 s by small 5–10 cm steps of the penguins (Fig 1C,1E, Movie S2, S3), reminiscent of a temporary fluidization [6]. These steps are also spatially coordinated and travel as a directed wave with a speed of ∼12 cm/s through the entire huddle (Fig. 1E). After the wave has reached the end of it, the huddle re-enters the jammed state. Interestingly the propagation speed of the traveling wave is comparable to the speed of the individual penguins during the step. This is analogous to the propagation of sound waves in an elastic entropic medium (gas or fluid) where typical molecular velocities are comparable to the velocity of pressure waves.

## Discussion

We propose that the small, regular steps serve a three-fold purpose. First, they help achieve the highest packing density. As new penguins join the huddle at the periphery, the small steps compact the huddle similar to the tapping of a bag of loosely packed granular material. Second, the small steps lead to a forward motion of the entire huddle. In addition, huddle movements allow separate smaller huddles to merge into larger clusters. Such merging is analogous to the merging of magnetic domains as the thermodynamic temperature is decreased towards the Curie point, the temperature above which a magnet loses its magnetism, or analogous to a phase transition in a disordered material that is brought towards a critical point. This is an essential process in condensed matter physics, penguins included. In further support of the phase transition analogy, we note that when the huddle breaks up, it occurs very rapidly [7], similar to the sharp jump in densities between e.g. a gas and liquid state. Third, the small repetitive steps lead over time to a slow macroscopic huddle reorganization. The nearly hexagonal packing arrangement of neighboring penguins in the inner region of the huddle is not disturbed by the traveling wave. In general, individual penguins do not change their positions relative to their neighbors, and they do not force their way in or out of a huddle. The time a penguin spends inside a huddle is determined foremost by the lifetime of the huddle, typically on the order of hours [5], and to a lesser degree by a treadmilling-like turnover of penguins that join the huddle preferentially at the trailing edge and leave the huddle at the leading edge.

Structural order of penguin positions in the huddle and coordinated movements arise in unison. A similar transition from individual to collective motion above a critical density has also been observed in other biological systems including marching locusts [8], tissue culture cells [9] and fish schools [10]. Such behavior resembles the fluid-to-solid gelation of short-ranged attractive colloids [11]. Gelation results from kinetic arrest due to crowding, whereby the particle motion is greatly reduced by the attractive interaction potential and the surrounding cages formed by the dense neighboring particles. In analogy, the huddling of penguins is triggered when the magnitude of the attractive interaction potential increases as the ambient temperature falls. The intermittent traveling waves help to speed up the relaxation of the penguin positions within the huddle towards an equilibrium configuration with hexagonal packing.

In this connection, it is noteworthy, first, that the gelation of weakly attractive colloids is similar to the colloidal glass transition of repulsive particles at high packing densities [11], and second, that repulsive interactions in active crowded systems such as humans during an escape panic are often accompanied with traveling waves [12]. Why these waves are uncoordinated, turbulent and dangerous in a human crowd but not in a penguin huddle remains an open question but may possibly depend on the shape and magnitude of the interaction potential, and on the distance of the system from an effective temperature characterizing a critical point. It is also unclear whether the traveling wave in a huddle is triggered by a single or few leading penguins and follows a well-defined hierarchy among group members, similar to the collective behavior in pigeon flocks [13], [14]. Modeling attempts with self-driven agents have explained collective behavior such as temporal and long-range spatial synchronization in bird flocks, fish schools or traffic congestion by evolutionary strategies and a small set of simple interaction rules between neighboring agents [15]–[20]. Similar mechanisms may also apply to the collective behavior of penguins in a huddle.

Taken together, these questions and observations link integrative biological functions to the macroscale dynamics of the underlying elements, and represent an unexpected intersection of topical issues in condensed matter physics and systems biology [21].

## Supporting Information

Movie S1 **Huddle formation and occurrence of coordinated traveling waves.** Time lapse recordings (full field of view) over 2 h (resolution reduced from 10 MP to 480 p), showing about half of the penguin colony during the aggregation and huddling process. At the beginning of the movie (∼12 p.m. with temperatures above −35°C), only few penguins aggregated in smaller huddles. As the temperatures gradually fell, larger and more stable huddles formed until nearly all the penguins aggregated in one large huddle.
(MP4)

Movie S2 **Huddle formation and occurrence of coordinated traveling waves (detail).** Time-lapse recordings (detail of S2 over 1 h) showing multiple huddles. The penguins in a huddle mostly face in the same direction which defines a rear end and a front end of the huddle. When a penguin joins the huddle, it does so by aligning itself first in the direction in which the other penguins are facing, and then moving closer to the huddle. As a result, penguins tend to join a huddle at its rear (trailing) end and leave it at the front (leading) end. During the periodic traveling wave, the huddles move in the forward direction (in the direction in which the majority of the penguins are facing).
(MP4)

Movie S3 **Coordinated traveling waves in a densely packed huddle.** 21 min sequence from S2 (detail corresponding to Fig. 1B) at reduced speed. The movie shows the travelling wave of small steps every 30–60 sec.
(MP4)
